# Improving standard of pediatric surgical care in a low resource setting: the key role of academic partnership

**DOI:** 10.1186/s13052-020-00827-2

**Published:** 2020-06-09

**Authors:** Pierluigi Lelli Chiesa, Osman T. M. Osman, Antonio Aloi, Mariagrazia Andriani, Alberto Benigni, Claudio Catucci, Paolo Giambelli, Gabriele Lisi, Faisal M. Nugud, Paola Presutti, Viviana Prussiani, Vincenzo Racalbuto, Fabio Rossi, Giuliana Santoponte, Bruno Turchetta, Diaa Eldinn Yaseen Mohammed Salman, Francesco Chiarelli, Alessandro Calisti

**Affiliations:** 1 Cattedra e UOC di Chirurgia Pediatrica – Università “Gabriele d’Annunzio” Chieti-Pescara, Via Fonte Romana, 8, 65124 Pescara, Italy; 2 University of Gezira, Wad Medani, Sudan; 3 Dipartimento di Medicina e Scienze dell’Invecchiamento, Università “Gabriele d’Annunzio”, Chieti-Pescara, Italy; 4 Ada Manes Foundation For Children, Wad Medani, Italy; 5 Dipartimento di Emergenza, Urgenza e Area Critica - A.S.S.T. “Papa Giovanni XXIII”, Bergamo, Italy; 6 Advising And Engineering Services, Roma, Italy; 7 Agenzia Italiana Cooperazione Allo Sviluppo, Sede di Khartoum, Wad Medani, Sudan; 8 Gezira National Center Of Pediatric Surgery, Wad Medani, Sudan; 9 Anestesia e Rianimazione, A.O. “San Camillo Forlanini”, Roma, Italy; 10 UOC di Chirurgia Pediatrica, A.O.U. “Maggiore della Carità”, Novara, Italy; 11 Anestesia e Rianimazione, P.O. “Santo Spirito”, Pescara, Italy; 12 Fondazione “Mangiagalli”, Milan, Italy; 13 Clinica Pediatrica, Università “Gabriele d’Annunzio”, Chieti-Pescara, Italy; 14 Chirurgia Pediatrica, A.O. “San Camillo Forlanini”, Rome, Italy

**Keywords:** Academic partnership, Pediatric surgery, Low resource setting, Sudan

## Abstract

**Background:**

An epidemiological transition is interesting Sub-Saharan Africa increasing the burden of non-communicable diseases most of which are of surgical interest. Local resources are far from meeting needs and, considering that 50% of the population is less than 14 years of age, Pediatric surgical coverage is specially affected. Efforts are made to improve standards of care and to increase the number of Pediatric surgeons through short-term specialist surgical Missions, facilities supported by humanitarian organization, academic Partnership, training abroad of local surgeons. This study is a half term report about three-years Partnership between the University of Chieti- Pescara, Italy and the University of Gezira, Sudan to upgrade standard of care at the Gezira National Centre for Pediatric Surgery (GNCPS) of Wad Medani. Four surgical Teams per year visited GNCPS. The Program was financed by the Italian Agency for Development Cooperation.

**Methods:**

The state of local infrastructure, current standard of care, analysis of caseload, surgical activity and results are reported. Methods utilized to assess local needs and to develop Partnership activities are described.

**Results:**

Main surgical task of the visiting Team were advancements in Colorectal procedures, Epispadias/Exstrophy Complex management and Hypospadias surgery (20% of major surgical procedures at the GNCPS). Intensive care facilities and staff to assist more complex cases (i.e. neonates) are still defective. Proctoring, training on the job of junior surgeons, anaesthetists and nurses, collaboration in educational programs, advisorship in hospital management, clinical governance, maintenance of infrastructure together with training opportunities in Italy were included by the Program. Despite on-going efforts, actions have not yet been followed by the expected results. More investments are needed on Healthcare infrastructures to increase health workers motivation and prevent brain drain.

**Conclusions:**

The key role that an Academic Partnership can play, acting through expatriated Teams working in the same constrained contest with the local workforce, must be emphasized. Besides clinical objectives, these types of Global Health Initiatives address improvement in management and clinical governance. The main obstacles to upgrade standard of care and level of surgery met by the Visiting Team are scarce investments on health infrastructure and a weak staff retention policy, reflecting in poor motivation and low performance.

## Introduction

Sub-Saharan African countries are witnessing an “epidemiological transition” due to the increasing proportion of Non-Communicable Diseases (NCD). In fact, in comparison to infectious diseases, metabolic, neoplastic, cardiovascular, congenital conditions and trauma account for about 50% of the total burden of diseases in Africa [1, 2]. Actually, NCD cause about a quarter of the deaths and disabilities among people living in low-income countries (LIC). Although diseases requiring surgery are highly represented among them [3] Pediatric Surgery in LIC is not developed [4, 5], despite more than 45% of the population is less than 14 years of age.

In high-income countries (HIC) pediatric surgeons are one for every 100,000 children under 15 years of age. In Africa, there is one pediatric surgeon for nearly 6,000,000 children [6] to deal with a significant burden [7] of congenital malformations and of acquired diseases, mostly of traumatic origin [8]. The development of Pediatric Surgery in LIC finds several obstacles in a reduced access to care due to poverty associated with shortage of health services and in lack of transportations that carries with it a risk of late referral for many life-threatening congenital conditions [9, 10]. Few specialists are concentrated in major cities, devoting much of their time to private practice and leaving rural areas uncovered. Better salaries and professional opportunities abroad attract many of them.

An increasing number of international initiatives have been developed in the last ten years to extend Pediatric Surgery in LIC through voluntary long and short-term humanitarian outreach initiatives.

This study describes an on-going program addressed to strengthen Pediatric Surgery in a Sudanese Tertiary Center by an academic bilateral partnership. Challenges, constraints, failures and accomplishments, met by the visiting teams are reported.

## Setting

A Memorandum of Understanding was signed in 2014, between a representative of the Italian Association of Pediatric Surgeons (SICP), the Ministry of Health of the State of Gezira and the University of Gezira to establish a partnership between an Italian Academic Institution and the Gezira National Center of Paediatric Surgery (GNCPS) of Wad Medani. It was addressed to upgrade the standard of surgical care for some selected diseases, including Hirschsprung’s disease (HSCR), Anorectal Malformations (ARM), bladder exstrophy epispadias complex, hypospadias. In 2017 this partnership was included in a three-year program implemented by the University “Gabriele d’Annunzio” of Chieti-Pescara, Italy, and financed by the Italian Agency for Development Cooperation (AICS). Italy is one of the largest donors in the Sudanese Healthcare Sector; in 2017 24 initiatives were implemented with an investment of 62% of the invested funds. The programs were developed not only in Primary Health Care (PHC), but also toward NCD.

Sudan, with a total area of 1,886,000 square kilometers is the third-largest country in Africa. The population was about 40 million in 2017, 45% under 14 years of age. The annual growth rate is 2.48%, life expectancy at birth 61.83 years, the incidence of poverty is 46.5%, and a per capita income is 1270 USD. [Source: UNDP, Sudan, http://www.sd.undp.org/content/sudan/en/home/ countryinfo/]. Pediatric surgery in Sudan was first recognized as an independent medical specialty in 1972, and currently, there are 14 pediatric surgeons in the entire country. Eleven of them are based in Khartoum hospitals, and three are serving outside the Capital. The Gezira State is located in Central Sudan, just Southeast of the confluence of the Blue and White Nile Rivers at the city of Khartoum, in the middle of the “Gezira scheme” the most massive irrigated scheme in Africa, founded in 1925. Wad Medani is the Capital. The State has an estimated multi-ethnic population of 4 million people. The University of Gezira was established in 1975 and included a Medical School. In Gezira, Pediatric Surgery started in 1983 as a small unit in the general Pediatric Hospital. The Ministry of Health of the Gezira State and the University of Gezira established in 2008 the Gezira National Centre for Pediatric Surgery (GNCPS) to provide facilities for children from Sudan and the neighbour sub-Saharan Countries.

## Materials and methods

### Resources

A deep insight was given into the GNCPS infrastructure, diagnostic facilities, technical equipment, medical, and nursing human resources, operating policies, to assess the current standard of care and main constraints. Access to care, Hospital financing system, resources management, and clinical governance were also evaluated.

### Admissions, case-mix, operative case volume, standard of care

Data were extracted from the last Hospital records available in 2014, on number of admissions and operations performed in both the elective and emergency settings before the current program was started.

### Partnership activities

The present paper reports cases observed, investigated, and operated by the visiting team in full cooperation with local surgical staff and their outcome. Training and teaching activities are illustrated. Medical pieces of equipment and educational supplies as well as the actions to promote clinical governance and better hospital management are also reported.

## Results

### Resources

#### Infrastructure

The GNCPS appears as a three-floors building with internal and external signs of deterioration and lack of maintenance. It includes outpatient clinics, Day Surgery unit, Medical lab, X-Ray & Ultrasound, four theatre rooms, laparoscopy room, three-bed hospital rooms, nursery with 8 incubators, general and private wards, lecture room with audio-visuals, medical statistics, medical secretariat, residents. The elevator to transport patients and materials to upper levels has never been installed, due to water leakage in the elevator shaft. The Hospital is hosting a mono-specialist activity (in this case, Pediatric Surgery) without any in-house allied clinical branch for multidisciplinary clinical management of cases.

#### Imaging

A Radiology Section is on the ground floor. The Remote-Control X-ray Equipment has not a technician to operate it on a 24-hours base and most patients of the GNCPS, even in critical conditions, must be manually transported to the nearby Children’s Hospital for any radiological investigation. A mobile X-ray machine is packed and cannot be moved to the upper floor to be used in the theatre or the wards. Modern ultrasonography equipment is also available, but no one in the Hospital can use it.

#### Pathology

There is no Pathology Laboratory in the Hospital, and specimens are sent to other Institutions. Frozen sections and special stains are not routinely available.

#### Laboratory

Many laboratory tests and genetic studies must be required in other Hospitals. A microbiology laboratory is available for Gram stain and culture/sensitivity. Hormonal and genetic workup for endocrine abnormalities is not always available.

#### Operating theatre

It includes four operating rooms and one sterilization room on the first floor. A Theatre for Emergencies is on the ground floor near to the Admissions Unit. The main critical issues found by the first Team were: no change rooms for medical and nursing staff with conveniences; no transfer bay for patient, material, and equipment; a lack of separation between aseptic area and clean and disposal areas; unrestricted scrub area without any elbow operated tap and soap dispenser; defective maintenance of equipment; insufficient cleaning, disinfection, inspection, packaging, and sterilization of surgical instruments; essential equipment for laparoscopy and cystoscopy is inaccurately stored and maintained; a 9 Fr. Pediatric Cystoscope cannot be used since optics (0° and 30°) have been severely damaged.

#### Anaesthesiology equipment

Cylinders dispense medical gases after the medical gas pipeline system has been disconnected and never reassembled. The cost of recovering the system is not sustainable by the Hospital. Modern ventilators and Monitors are available, but flow meters, filters, circuits, and probes are frequently missing.

#### Critical care

A four beds recovery room, adjacent to theatre, lack of trained nurses. Some syringe-pumps are available, but disposable infusion sets are not in store. Three monitors are in use, but connecting cables and probes for different age groups are often not available. There is no automatic or semi-automatic defibrillator or cardioline in the Hospital. Hemogasanalysis equipment is available at the nearby Pediatric Hospital.

#### Patients wards

are on the ground and second floor. From fifty to sixty beds are distributed among one large common area of 25 beds, two large rooms for seven-bed each and 5 two beds rooms. There is a Neonatal Unit with five incubators of different brands and models with an unreliable temperature and humidity control system. Patients’ identification cards at the bedside are not used; bed sheets are reserved for the recovery room only. Works are in progress to make an isolated treatment room for invasive procedures (blood sampling, catheter insertion, dressing), which are currently done at the bedside where sterility is obviously not guaranteed.

#### Health care providers

There are forty-five nurses in service at the GNCPS, including fifteen recently graduated that still require adequate training. They work on two shifts: 7 am – 2 pm and 2 pm – 7 am. Many of them usually wear traditional clothing, which makes identification of their role sometimes impossible. A matron coordinates the ward staff and there also is a head nurse for theatre staff. Scrub nurses rotate either in assisting the surgeons in scrubbing in for surgery and preparing the operating room by maintaining a sterile procedure to set out instruments and set up other surgical equipment. They also take care of instruments, decontamination, cleaning, sterilization, and packing. Low level of motivation in the nurses of most the under-resourced countries, as described by Oshvandi K et al. [11], is a significant problem also at the GNCPS. It is mainly related to lack of supportive supervision by matrons and consultants and job difficulties due to poor infrastructure and equipment, low wages, lack of authority and recognition, associated with physician-centered hospital culture.

One anaesthesiologist, who supervises twelve anaesthesiology technicians, has been recently appointed and assigned to the operating theatre but with some duties to cover at the Gezira Cardiac Centre, whenever procedures on children are scheduled. At the GNCPS, anaestesiologists are not responsible for postoperative care even on critical patients as technicians are not provided with medical skills making them able to assist patients outside of surgery. Operated patients are usually followed in the recovery room by the on-duty surgical and the on-duty nursing staff.

The GNCPS is included in the University of Gezira Pediatric Surgical Residency Program, attended by twenty-seven residents per year. The current general pediatric surgery curriculum incorporates rotations in all surgical specialties. Registrars in training provide general surgical services and make scheduling of patients for surgery under the supervision of consultants. They also admit patients from the Emergency Department, attend daily ward rounds with the surgical team, take hand-over of patients from previous shift registrar so that continuity of care is ensured and immediate decisions are taken. Orders are written. They also review patients regularly following the plan of care, inform the consultant of any issues or problems and cover operating room duties. Registrars are very active and interested in surgical procedures and show excellent “technical skills” and theoretical background. Senior registrars cover duty shifts and take responsibility for any emergency procedures. Unfortunately, supervision by the few consultants is not always as it should be expected in a teaching Hospital like the GNCPS. Main aspects of the preoperative diagnostic workup, communication skills with patients’ families, postoperative assistance, treatment, and follow up, are sometimes neglected. Postoperative management is left to nursing staff, but they are rarely kept in due consideration and adequately informed about patients’ procedures and their implications. Surgeons lack familiarity with the use of imaging techniques and rely mainly on history and clinical features that carry a high risk, for the junior members of medical staff, of mistreating complex cases.

#### Hospital management and clinical governance

A consultant is appointed as Director of the Centre, assisted by an administrator. There are two more consultants, both involved in clinical activities, teaching, and tutoring. Responsibilities and decision-making procedures about key critical clinical systems and processes are not clearly defined. No Hospital Management Board, including representatives of other Health professionals, is established. The Ministry of Health of the State of Gezira provides for payment of staff salaries only. Patient's families are requested to provide themselves with medical devices and drugs needed during the Hospital stay, except for those covered by an insurance, which pays Hospital fees according to a rate usually lower than real hospital costs. Maintenance, refurbishing, and renewal of equipment, when necessary, depend mainly on the small investments by the local State Government.

#### Admissions, case-mix, operative case volume, and standard of care

From January to November 2014, about 7000 admissions and 3374 Operations (897 major procedures, 26%) were recorded at the GNCPS. Fifty-seven percent of them were on emergency. Data on admissions, case mix and the outcome are not easy to obtain so far and must be derived from hand-written logbooks. Personal data and addresses are sometimes incomplete, making recalling for follow up often impossible. A high number of procedures are reserved for congenital hydrocephalus and meningocele. Spina bifida and its neurological and urological complications still affect a relevant number of Sudanese neonates. Pediatric urological surgery is limited to procedures to remove stones, very common in the area. Pre and perinatal ultrasonography screening is not available, and congenital urinary tract anomalies are commonly missed in early life and are later managed by adult urologists, when symptomatic. The most common urological conditions observed at the GNCPS were hypospadias (70 cases per year) and cryptorchidism (126 cases per year). Hypospadias cases are sometimes referred after a failed correction by inexperienced adult surgeons [12]. Inguinal hernia/hydrocele (210 cases/year) was the second most frequent discharge diagnosis after appendicectomy (237 cases/year). Pyloromyotomies for pyloric stenosis was reported to be only 24 cases per year. An outreach program was recently started to deliver pediatric surgical services in some peripheral general hospitals to reduce the workload of minor surgery on the GNCPS and to offer a better service to patients living in remote rural areas [13]. Sporadic cases of solid abdominal tumors eligible for resection were recorded. Adjuvant chemotherapy is available at Wad Medani by a Pediatric Oncology Service at the Oncological Hospital [14].

Surgery for the most common neonatal conditions seen in western countries, like oesophageal atresia, omphalocele/gastroschisis, small bowel obstruction and diaphragmatic hernia is rather occasional at the GNCPS (35 cases/year). These newborns are usually late referred or die before they are diagnosed. In Sudan most of the deliveries do not occur in hospitals. Early detection of congenital defects is not warranted for these babies as for those born in a health care facility. No guidelines to be shared among obstetricians, midwives, and neonatologists exist on safe referral to pediatric surgeons. Most of neonatal cases that survive long enough to be diagnosed are sent from the GNCPS to Khartoum for treatment, since a Neonatal Intensive Care Unit for critical surgical cases is still not available.

**Fig. 1:**
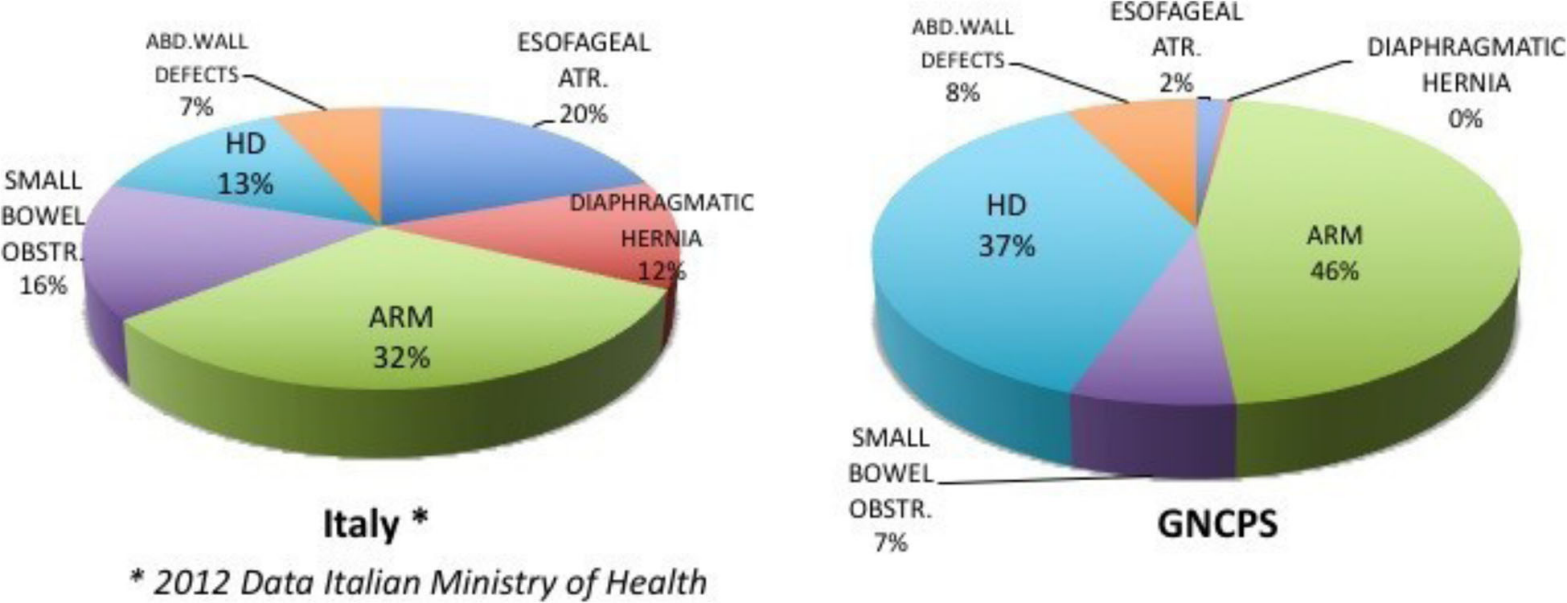
Differences in the incidence rate of main congenital abnormalities of surgical interest in a Western Country and at the GNCPS

As reported from all Sub-Saharan countries [15–17], ARM and HSCR cases may result, so far, overrepresented among neonatal congenital diseases compared to data reported from Western Countries. Figure 1 brings together data collected from the GNCPS and Italian Pediatric Surgical Centres. *[**http://www.salute.gov.it/portale/temi/ric_codice/default.jsp**]*.

Female patients with vestibular/vaginal fistula or cloaca not associated with intestinal obstruction remain sometimes undiagnosed until adolescence. Survival of males with a recto-urinary fistula is possible after an emergency colostomy which is often done in rural hospitals with an improper technique and is frequently associated with complications (prolapse, stenosis). Stomas are not always well accepted by families living in rural areas and frequently they do not receive proper care; stoma bags are not affordable by most people and peristomal skin lesions, bleeding colonic mucosa and ulcers are sometimes observed. A long time may be required before these patients can benefit from a definitive treatment by a perineal anorectoplasty (PSARP) which is done without the assistance of an electric muscle stimulator and is frequently followed by incontinence or by a stenosis due the irregular follow up and lack of a dilatation regimen (17) as in many African reported series.

As far as HSCR is concerned, frozen sections and special stains are not routinely available, and there is no experience of suction rectal biopsies [18]. A clear cut distinction between HSCR and primary constipation is not always possible in acute presentations, due to the low level of radiological imaging [19]. A leveling colostomy is the first option, followed by a secondary Swenson pull through of the upper stoma, possibly after a transanal biopsy. The risk of surgical overtreatment of constipation is consequently high. Many patients with bladder exstrophy are seen at the GNCPS, commonly referred late. A primary reconstruction is an attractive perspective for the cases seen in the firsts months of life. This challenging procedure, including pelvic osteotomy, and requiring a prolonged postoperative immobilization under sedation and a meticulous nursing is not possible at the GNCPS, as in many in under-resourced Hospitals of Sub-Saharan Africa [20–22]. Some visiting pediatric urologists, supported by charities, tried primary closure in many infants followed, in most cases, by partial or total disruption of bladder wall and neck associated with a pubic bone dislocation. In the few successful cases, continence remained a problem, and upper urinary tract control was commonly omitted [22].

### Visiting team activities and results

The Program activities were inspired by the:

- Global Goal 3 “To ensure healthy lives and well-being for all”, to contribute reducing the disparity in access to care of children affected by diseases of surgical interest in low resources countries and to reduce the impact of disabilities related to correctable congenital diseases.
- Addis Ababa Action Agenda (AAAA) Science, Technology, Innovation, and Capacity-Building Section, where it calls to “resolve to adopt science, technology, and innovation strategies as integral elements of national sustainable development strategies to help to strengthen knowledge”.

The Program aimed to set a better standard of surgical care at the GNCPS by training on the healthcare providers and included assistance in clinical governance and hospital management. Increasing the number of new specialists in paediatric surgery is a primary goal for African Countries, but special attention was also given by the visiting teams to those registrars in general surgery rotating at the GNCPS. They all must achieve the necessary skills to deal safely with the most common paediatric surgical conditions, in district and rural hospitals and to refer more difficult cases to a Specialist Center. Training on the job of anaesthesiologists and nurses’ knowledge were also cared.

### Surgical training on the job and proctoring

from October 2017 to February 2020 eleven Teams traveled to Wad Medani from Italy working for a total of thirty-three weeks at the GNCPS. Ten of them included surgeons, nurses, and anaesthesiologists and one team included one anaesthesiologist and five nurses in charge of teaching and tutoring without any direct clinical commitment. Seven pediatric surgeons, five anaesthesiologists, fourteen nurses from Italy rotated at the GNCPS, participating in all Hospital activities, ward round, surgical sessions, clinical meetings, tutorials, and academic lectures. The team members came from some paediatric surgical centres in Italy and were supervised by the head of the Paediatric Surgery Unit of the University of Chieti-Pescara as the clinical and scientific director of the program. The visiting nurses’ job was to train on the job local ward and theatre staff on pre- and post-operative care, safe surgery and infection control. Visiting anaesthesiologists worked with their local technical partners to update current protocols and practices. The anaesthesiologist who was recently appointed following the advice of the Italian Visiting Team (IVT) shared these tasks. Based on the original Memorandum of Understanding, surgeons of the visiting team focused on improving the management of some more frequent and severe conditions. It is the case of ARM management practices starting from colostomy and including preoperative nursing, PSARP technique and follow-up were revised. Differential diagnosis of constipation and HSCR surgery, operative procedures for complex or re-do hypospadias, sustainable alternatives to primary closures in bladder exstrophy/epispadias complex were mainly focused. All these conditions represent 20% of all major procedures at the GNCPS.

Visiting surgeons examined 204 complex cases along with 30 weeks of clinical activity (Table 1). A referral to a better-equipped centre was decided together with local consultants for twelve patients due either to lack of facilities to guarantee a safe postoperative course or to missing equipment to consent a thorough radiological assessment. Two hundred and sixteen procedures were performed, and sustainable surgical solutions were always preferred and recommended. One hundred and nine procedures have been scheduled for future missions. All surgical indications were discussed with local consultants and registrars. Table 2 shows the impact of corrective measures adopted at the GNCPS to achieve a better surgical outcome; a group of senior registrars regularly assisted visiting surgeons in the diagnostic workup of new cases and the in the theatre work. They acted as the first operator in 30-50% of major surgical cases, under the supervision of visiting surgeons. The present program included, every year, also a three months visit of one consultant from the GNCPS to the Paediatric Surgery Unit of the University of Chieti-Pescara at the Pescara General Hospital to observe hospital service organization, governance, and management and take part to clinical meetings and hospital audits where treatments are discussed and revised. In 2018, the Director of the GNCPS granted this fellowship.

**Table 1:** Major cases observed and treatment performed or planned by the Visiting Team from October 2017 to October 2019 (21 weeks)

	No	M/F ratio	Type	Procedures performed & scheduled
**ARM**	**94**	47/47	34 Recto bulbar fistulae 4 Recto bladder neck fistula 2 Recto prostatic fistula 5 Rectoperineal fistula 2 Rectal atresia 2 no fistula 7 Persistent Cloaca 36 Rectovestibular fistulae 2 Recto vaginal H fistula	57 PSARP (4 re-do, 2 TUGM) 24 Colostomies (12 re-do) 2 Malone conduits Scheduled: 31 PSARP (2 re-do), 11 Colostomies (5 re-do)
**Suspected HD**	**17**	16/1		2 De la Torre pull Through, 1 Malone conduit scheduled Scheduled after pathology report: 4 De la Torre pull Through, 1 Malone conduit scheduled
**Hypospadias**	**48**		16 proximal 12 Midshaft 20 distal	27 one step procedures (6 re-do), 25 staged procedures Scheduled: 11 primary treatment, 5 secondary treatment
**Exstrophy-Epispadias complex**	**26**	20/6	18 Exstrophyc Bladders 8 Epispadias	4 Mitchell penile disassembly, 6 Mainz II Pouches Scheduled: 4 Mitchell penile disassembly, 7 Mainz II Pouches

**Table 2:** Quality Assurance of Surgical Standards for selected complex pediatric procedures

GNCPS Major Surgical Procedures/year *(877 out of total 3374 operated cases in 2014)*		Main critical issues found.	Corrective measures	Results achieved or expected within a short term
***Exstrophy/Epispadias Complex repair***	***20% among Major procedures***	*Too frequent late referral* *Poor continence after primary Bladder closure.* *Poor quality of life* *Follow up facilities unavailable.*	*Internal Continent Urinary Diversion (Mainz II) vs Primary Bladder Closure in late referred cases, failures after primary closure*	*Faster postoperative recovery after Mainz II* *Reduced complication rate* *Continence always achieved in short term* *Better quality of life* *Easier follow up*
***Hypospadias repair***		*Recurrent failures of midshaft & proximal Hypospadias management*	*On the job Tutorials*	*Reduced number of failures*
***Divided Colostomy***		*37% stoma confection by Residents unsatisfactory (prolapsed, inverted, retracted, poor sited)*	*On the job training of Residents* *on Colostomy confection* *Re-do of failures*	*Residents performing 50% of new colostomies under Visiting Surgeons assistance with better results*
***PSARP for ARM & CLOACA***		*pre-op distal loopgram Inadequate* *poor distal loop preoperative cleaning* *PSARP procedures without nerve stimulator* *>60% post-op incontinence* *frequent post-op wound infections* *Post-op anal stenosis secondary to unattended dilatation regimen*	*on the Job training on loopgram and distal loop pre-op cleaning* *Nervous stimulator supplied* *20 sets of Hegar dilators donated* *post-op Bowel Management introduced* *Malone conduit (MACE) for post PSARP incontinence*	*Residents supervising all distal loopgrams and distal loop pre-op cleaning* *All PSARP were done under nerve stimulator assistance* *Mothers, trained to do post-PSARP dilatation, paying a rental deposit to take home the dilators set* *Post-PSARP follow up clinic established with a Staff Nurse trained to Bowel management*
***Surgery for H.D.***		*X-ray imaging unreliable* *High risk of inappropriate pull through for Constipation without H.D.* *Suction rectal biopsy and Frozen section biopsy unavailable.*	*Prevention of surgical overtreatment of constipation* *Suction rectal biopsy device donated* *Tutorials on Radiology for H.D. Transanal de La Torre Pull-Through*	*Residents trained to perform Suction rectal biopsies in all cases of suspected H.D.* *The pathologist learning curve on suction biopsy specimens has started* *Residents supervising Barium enema for all suspected H.D.* *Visiting Surgeons assisting local Consultant doing De la Torre Pull-Through*

#### Anorectal malformations (ARM**)**

Ninety-four cases were referred to the visiting team at the mean age of 21 months. Forty-seven were male (34 recto-bulbar fistulae, 4 recto-bladder neck fistula, 2 recto-prostatic fistula, 4 recto-perineal fistulae, 2 rectal atresia, 1 no fistula) and forty-seven were female (7 persistent cloaca, 36 recto-vestibular fistulae, 2 H type fistula, 1 perineal fistula, 1 no fistula). Eighty patients came with an untreated ARM, and 66 of them had a divided stoma. Colostomy was inadequate in 25 patients (prolapse, inverted loop, stenosis), and re-doing was needed for 17 of them. Sudanese Registrars (either paediatric surgeons and general surgeons) performed new and re-do colostomies under close supervision. They were always reminded about the crucial, preliminary role of this operation for a successful anoplasty. A high-pressure distal loopogram was rarely done properly at the GNCPS. Low-pressure injection of contrast and presence of stools or fecal debris was responsible for missing recto-urinary fistula in males. Fourteen referred cases had already a PSARP procedure, which had been followed by a poor outcome or sequelae; a re-do was feasible in 9 of them only. A PSARP was done in 57 patients (including three re-doing and two persistent cloaca cases with a total urogenital mobilization–TUGM) and was planned in 31. Visiting surgeons assisted local registrars performing one-quarter of them. Since a muscle stimulator was donated to the GNCPS, in 2018, no PSARP was done without it. Surgical site infections occurred in 9 patients and wound toilet/revision under anaesthesia of perineal wounds were needed. Despite meticulous preoperative cleaning and wash out of the distal loop before PSARP were always recommended, results were not always satisfactory either for the scarce protection offered by some unsuitable colostomies either for the low level of postoperative nursing care; visiting nurses thoroughly trained local staff on how to prevent these complications. Some patients were lost from follow up after preliminary colostomy and postoperative dilatations program suffered from the lack of systematic follow-up after PSARP; colostomy closure was possible in 46% of the cases until now. These problems were mainly due to difficulties in travelling and accessing hospital care among poor people living in rural areas. Two severe anal stenosis were observed among unfollowed patients.

#### Hirschsprung disease (HSCR)

Seventeen children were referred to the visiting team for a suspected HSCR. Three had a previous Swenson pull through, which resulted in incontinence in two cases (both accepted a Malone conduit procedure for antegrade enema) and in persistent constipation in another one for a presumable residual aganglionic bowel segment. Eight cases (mean age three years) presented a leveling “loop colostomy” done on the basis of the clinical history and presentation. Both presented a huge fecal impaction in the distal loop. The visiting surgeons suggested a transanal De La Torre [23] pull through of the stoma site in two of these cases where histological HSCR final diagnosis was confirmed and assisted the local consultants performing this procedure for the first time; both cases had a favourable outcome. The other five patients are waiting for the same procedure in a short time. Other six children (mean age three months) came with a history of delayed meconium emission and severe constipation. The visiting team introduced at the GNCPS suction biopsy device for these new cases. Routine multiple colonic seromuscular biopsies were also always recommended to local surgeons whenever the abdomen of a constipated child was explored on emergency and a levelling colostomy was planned.

#### Hypospadias

Forty-eight cases (range 6 months-15 years, mean age 5.8 years) were seen. Twenty of them (41%) were distal hypospadias; twelve cases were a midshaft form and sixteen (33%) were proximal or perineal hypospadias, all with a severe chordee. This high percentage of complex, proximal types was due to the special opportunity offered to the GNCPS to submit these challenging cases to the visiting team. Ten of them had at least one previous failed treatment and six showed benefit from a re-do operation (four to close a fistula), which was always performed together with the local consultants. Fifty-two operations in total were done on 37 patients: a multiple steps procedure was required in ten, in six Bracka a urethroplasty was done; fistula developed in five operated cases and required a second procedure; eleven patients are still waiting for primary treatment; five secondary procedures are on the waiting list.

#### Exstrophy-Epispadias complex

The visiting team examined 8 incontinent epispadias cases (one had a previously failed treatment at the GNCPS). In four of them, a Mitchell's successful procedure was done by the Visiting Team, and four are waiting for treatment. Eighteen cases of bladder exstrophy (12 boys and 6 girls) were also seen; in seven, primary closure had been attempted at the GNCPS followed by a total or partial disruption; eleven cases were referred to at the mean age of 5 years (range 1 months-12 years) with an untreated anomaly. Unfortunately, the GNCPS cannot offer a safe context for a primary bladder closure. Adequate postoperative traction and immobilization, pain and sedation management, nutritional management, advanced nursing care cannot be guaranteed. The only option for five cases under two months of age was the referral to a Pediatric Surgical Unit in Khartoum, where these facilities are available. For all the other 13 patients (M/F 10/3 mean age 5.8 years, range 1 yr. - 12 yrs.), a Mainz II internal diversion appeared to be the option more compatible with the local context. It was introduced at the GNCPS for the first time and already been done in six children by local Consultants assisted by a visiting surgeon. These patients had all, at six months, a socially acceptable continence, a normal renal function and a not dilated upper urinary tract at the ultrasound.

### Equipment

A muscle stimulator to identify the muscle sphincter complex during PSARP procedures was donated, together with several Hegar dilators sets. A cooperative Sudanese nurse was trained to teach mothers performing anal dilatations themselves, according to a strict Bowel Management Program [24]. Each family received a complete dilator set before discharge from the Hospital under payment of a small deposit. Follow up visits were scheduled to assess time for colostomy closure on the basis of anal calibre. Supervision of the ARM patients follow-up by a Senior Registrar was strongly recommended but not yet started. A suction biopsy device for diagnosis of HSCR was donated to the GNCPS by the Program Steering Committee. A long learning curve is expected from the local pathologist before fully reliable results are available.

### Educational material

Copies of several Pediatric Surgery and allied sciences textbooks, in their most recent digital editions, were donated to all registrars on training at the GNCPS.

### Meetings-lectures

Medical members of the visiting team attended daily morning meetings and case sessions, taking an active part in the clinical discussion. Weekly lectures on selected topics were also given to registrars and students of the University of Gezira School of Medicine. Tutorials and PowerPoint slides were given for anaesthesia technicians and nurses, showing the most updated protocols and procedures. Particular emphasis was given during lectures and tutorials about the importance of imaging in the diagnostic pathway of pediatric surgical diseases. An advanced course on single surgical topics (e.g., colorectal surgery, hypospadias, exstrophyc bladder) was arranged, when possible, inviting qualified experts and was extended to surgeons from other Sudanese regions. Program schedule included an annual workshop to improve awareness about the burden of pediatric surgical diseases at a community level and to enforce common strategies among Gezira State Health Providers and Stakeholders acting in the field of women and children’s health.

### Advisorship

In the first two years of the program, a technical team from Italy, including experts in the field of Hospital Management, Hospital Architectural Design, Medical Engineering, and Equipment Maintenance, visited the GNCPS infrastructure. They suggested some changes in the Hospital structure and equipment to improve the delivery of medical care, patients and staff satisfaction and to reduce related healthcare costs. The impact of appropriate architectural design is crucial in preventing healthcare-associated infections through a better partition of clinical spaces, that can be readily cleaned and decontaminated. The Gezira State Ministry of Health recently financed some of the works suggested by the technical team, like a new space partition of theatre and a new power supply station for the X-ray Department. The visiting team is supporting local management to introduce a new model of clinical governance at the GNCPS in order to promote teamwork and communication, to set new standards for processes and clinical guidelines, to encourage internal audit sessions, to establish a collaborative network within Maternal and Child Health Sector of Gezira State.

## Discussion

This review aimed to highlight main critical issues, challenges, and solutions adopted in the development of an Academic Partnership Program between an Italian University Centre of Paediatric Surgery and a similar Institute in Sudan. It was aimed to improve the standard of care in a Hospital setting constrained by limited resources, sharing experiences and knowledge and training on the job and teaching residents, anaesthesia technicians and nurses.

Modalities to address the burden of paediatric surgical diseases in low resource countries, as in Sub Saharan Africa, are still a matter of discussion. A large number of international initiatives, many of them unpublished, have been undertaken according to different intervention paradigms. There is a lively debate about the impact of these initiatives on immediate local health needs and the related investments in terms of capacity building of local providers. Disaster and crisis relief programs focus on the temporary deployment of highly equipped teams, but education is not within their scope [25]. Short term, self-contained, missions from qualified international groups and associations often focus on selected pathologies (eye, cleft lip, congenital heart diseases). They stress local infrastructure and have a high cost in terms of involved expatriated doctors and nurses. Although great relief has given to many children without other chances of care, they do not produce the improvement of local health resources apart from those needed for patient recruitment and follow-up [26]. Establishing a specialty hospital in a low resource context by international funding is also a challenging task.

It can be an educational opportunity for local health professionals, but requires long-term investments and, due to access limitations, its impact on the local needs is still low compared to high costs. Capacity building through academic partnership is an attractive modality of development cooperation [27] that inspired this program. Formal surgical training of Sudanese doctors in Italy was not considered in our initiative and it should have been under the strict rules for admission of foreign graduates to medical residency programs. Training abroad of graduates from LIC is a debated issue as it has a high cost and may encourage brain drain. Our partnership started with a multilateral agreement with local academic institutions about goals to be achieved. Measurable outcomes were defined to improve health standards, staff training and resources management. A continuous, frank and thought-out analysis of local needs and constraints informed our on-going relationship based on trust and respect [28]. Emphasis was put on proposing sustainable models of treatment and cultural issues affecting medical care in the host country were always considered [29]. Compromises on clinical results were never accepted on the basis that “doing something is better than doing nothing”. The role of the expatriated team was primarily educational. The number of surgeons, anesthesiologists and nurses rotating in the host Country was intentionally limited to enforce mutual trust with local staff and integration, friendship ties and mutual respect. Starting from the third year of the Program, only one surgeon and two nurses took part in each mission and local anaesthesiologists assisted the visiting team in surgical activities. Visiting surgeons taught on the job Sudanese Paediatric Surgical Registrars, who rotate for 6 months at the GNCPS and General Surgical Registrars, spending there two months of specialist training, to introduce them to different procedures gradually and to make able to perform them unsupervised. Indications to treatment were always discussed during morning meeting and comments on postoperative course and audit on outcomes were part of weekly afternoon sessions dedicated to workshops, lectures and tutorials on specific issues emerging from daily clinical activities.

Partnership clinical activities were focused on a group of diseases selected on the basis of the local epidemiological context providing training on the job of medical and nursing staff and upgrade of treatment protocols and current standard of care, especially for ARM. Infection control practices appeared still mostly unattended due to infrastructural problems and additional work has still to be done together by the visiting and the local nursing staff about this issue. A great effort was dedicated by the visiting teams to offer sustainable solutions for late referred cases of epispadias/exstrophy complex and to offer a technical update on the treatment of severe proximal hypospadias.

Despite multiple limits and constraints, the present program is having a favourable cost-benefit ratio and may have a significant impact on capacity building, compared to other modalities of healthcare development cooperation. Training on the job of hospital staff, mentoring the in-training medical residents, working side to side with local consultants, sharing knowledge and experience, introducing sustainable models for a better human, technological and infrastructural resource management and clinical governance might contribute to improve the standard of care in developing countries.

Obviously, all these efforts cannot compensate for lack of adequate structural investments, effective human resource policy and a comprehensive strategy on maternal and child health [30, 31]. There are many motivational factors influencing health care providers’ retention in low resource contests. They are primarily related to financial incentives but, although these remain a potent remedy to the “brain drain” that affects low resource countries, we cannot forget that motivations are also strengthened by the work context, like infrastructure, organization, supplies and human and professional recognition and respect.

## Conclusions

The key role that an Academic Partnership can play, acting through expatriated Teams working in the same constrained contest with the local workforce, must be emphasized. Besides clinical objectives, these types of Global Health Initiatives address improvement in management and clinical governance. The main obstacles to upgrade standard of care and level of surgery met by the Visiting Team are scarce investments on health infrastructure and a weak staff retention policy, reflecting in poor motivation and low performance.

## Data Availability

The datasets used and/or analyzed during the current study are available from the corresponding author on reasonable request.

## Abbreviations

AICS: Italian Agency for Development Cooperation
ARM: Ano-Rectal Malformation
GHI: Global Health Initiatives
GNCPS: Gezira National Centre for Pediatric Surgery
HIC: High-Income Countries
HSCR: Hirschsprung’s Disease
IVT: Italian Visiting Team
LIC: Low-Income Countries
MACE: Malone Antegrade Cleaning Enema
NCD: Non-Communicable Disease
NIC: Neonatal Intensive Care
PHC: Primary Health Care
PSARP: Posterior Sagittal Ano-Rectoplasty
SICP: Italian Association of Pediatric Surgeons
TUGM: Total Uro-Genital Mobilization
